# Reply to Del Riccio, M.; Lastrucci, V. Comment on “Maglione et al. Changes in Respiratory Viruses’ Activity in Children During the COVID-19 Pandemic: A Systematic Review. *J. Clin. Med.* 2025, *14*, 1387”

**DOI:** 10.3390/jcm14092960

**Published:** 2025-04-25

**Authors:** Marco Maglione, Vincenzo Tipo, Emiliano Barbieri, Roberta Ragucci, Agnese Sara Ciccarelli, Chiara Esposito, Ludovica Carangelo, Antonietta Giannattasio

**Affiliations:** Pediatric Emergency Unit, Santobono-Pausilipon Children’s Hospital, 80129 Naples, Italy; v.tipo@santobonopausilipon.it (V.T.); emiliano.barbieri@unina.it (E.B.); ro.ragucci@unina.it (R.R.); agnesesara.ciccarelli@studenti.unicampania.it (A.S.C.); chiara.esposito@unina.it (C.E.); l.carangelo@santobonopausilipon.it (L.C.); a.giannattasio@santobonopausilipon.it (A.G.)

We would like to thank Dr. Del Riccio and Dr. Lastrucci for the interest in our systematic review showed in their article entitled “Comment on Maglione et al. Changes in Respiratory Viruses’ Activity in Children During the COVID-19 Pandemic: A Systematic Review” [1] and for their constructive comments.

We appreciate their contribution to the field with the study “Seasonality and Severity of Respiratory Syncytial Virus During the COVID-19 Pandemic: A Dynamic Cohort Study” [2], which offers relevant and timely insights into the post-pandemic epidemiology of RSV. Their findings regarding the shift in RSV seasonality, increased incidence following the lifting of restrictions, and higher burden among older children are clearly aligned with the broader trends highlighted in our review.

As outlined in our methodology, our systematic search included studies published up to November 2024 and available in full text. Although we aimed to include early online publications indexed in databases such as PubMed and Scopus, it is possible that their manuscript, while available as an “in press” version, was not yet fully indexed or retrievable through our search filters at the time of data collection. This is an acknowledged limitation of systematic reviews, especially in rapidly evolving fields and during periods of intense publication activity.

We sincerely acknowledge the value of their study, which further reinforces the need for continued epidemiological monitoring and supports several key conclusions of our review. We are grateful for their engagement with our work, and we believe that such scientific dialogue contributes meaningfully to the advancement of pediatric infectious disease research.
